# Estrogen Signals through ERβ in Breast Cancer; What We Have Learned since the Discovery of the Receptor

**DOI:** 10.3390/receptors3020010

**Published:** 2024-05-03

**Authors:** Harika Nagandla, Christoforos Thomas

**Affiliations:** Houston Methodist Neal Cancer Center, Houston Methodist Research Institute, Houston, TX 77030, USA;

**Keywords:** ERβ, breast cancer, ERβ ligands, tumor progression, tumor microenvironment

## Abstract

Estrogen receptor (ER) β (ERβ) is the second ER subtype that mediates the effects of estrogen in target tissues along with ERα that represents a validated biomarker and target for endocrine therapy in breast cancer. ERα was the only known ER subtype until 1996 when the discovery of ERβ opened a new chapter in endocrinology and prompted a thorough reevaluation of the estrogen signaling paradigm. Unlike the oncogenic ERα, ERβ has been proposed to function as a tumor suppressor in breast cancer, and extensive research is underway to uncover the full spectrum of ERβ activities and elucidate its mechanism of action. Recent studies have relied on new transgenic models to capture effects in normal and malignant breast that were not previously detected. They have also benefited from the development of highly specific synthetic ligands that are used to demonstrate distinct mechanisms of gene regulation in cancer. As a result, significant new information about the biology and clinical importance of ERβ is now available, which is the focus of discussion in the present article.

## 1. Introduction

Estrogen receptor α (ERα) was first identified by Elwood Jensen in 1958 [1] and cloned from MCF-7 cells in 1986. ERα had long been considered as the sole mediator of estrogen signaling until the discovery of ERβ by the group of Jan-Åke Gustafsson in 1996 [2]. ERα and ERβ are encoded from different genes that reside on separate chromosomes. The ERα gene (ESR1) is located on chromosome 6q25.1 and encodes for a protein that is 595 amino acids long, whereas the ERβ gene (ESR2) locus is on chromosome 14q23.2 and gives rise to a 530 amino acid product [3]. As members of the nuclear receptor superfamily, both ER subtypes have three main functional domains: the N-terminal domain with activation function 1 (AF-1) that regulates target gene transcription independent of ligand activation, the DNA-binding domain (DBD) that facilitates binding to specific estrogen response elements (EREs) in regulatory regions of target genes, and the receptor dimerization and C-terminal ligand-binding domain (LBD) with AF-2 function. Despite the presence of common structural and functional domains, there is 95% and 59% homology in DBD and LBD between ERα and ERβ, respectively, indicating the diverse nature of ligands that bind to ER subtypes and the similarity in the mechanism of DNA interaction during the regulation of target gene expression [4]. Minor differences in LBD and low homology in AF-1 that determines the interactions with co-regulators point to a distinct mechanism of action by the ER subtypes in target tissues.

Because of the different function of ER subtypes, the nature of estrogen response in target tissues primarily depends on the availability of the receptor subtype and the presence of co-regulators and partner transcription factors that control the transcriptional activity. ERα is mainly expressed in the mammary gland, uterus, thecal cells in the ovary, bone, male reproductive organs (testes and epididymis), prostate stroma, liver, and adipose tissue. ERβ, on the hand, is predominant in granulosa cells of the ovary, the prostatic epithelium, bladder, colon, bone, lung, breast and adipose tissue as well as in the central nervous, cardiovascular and immune system [5–8].

Our knowledge about the biology of ERs has primarily been derived from their study in the reproductive system, mammary gland, and in breast cancer [9]. It is now well accepted that increased signaling through ERα is essential for the growth of the mammary gland during development and pregnancy but has oncogenic properties in breast cancer. About 70% of breast cancers overexpress ERα, indicating its importance as a molecular target as well as in prognosis of the disease. The induction of ERα signaling in luminal tumors has long been the focus of endocrine therapy, including the ERα antagonist tamoxifen that has represented the gold standard for treatment of ERα-positive breast cancer for over five decades. On the other hand, owing to challenges in detection due to decreased protein expression, the use of non-specific antibodies, and the contribution of alternatively spliced and differentially expressed isoforms to the immunohistochemical signal, initial reports of ERβ action and clinical importance in breast cancer were conflicting. Over two decades of extensive research using preclinical models and human specimens has reinvigorated interest in ERβ by uncovering effects that link the expression and function of the receptor to the biology of the disease. While ERβ is expressed in epithelial, myoepithelial and stromal cells of breast tissue during development and in adulthood, its levels have been reported to decline in breast cancer [10–14] due to oncogenic signaling that primarily represses the activity of the promoter of the ERβ gene [14,15]. The decreased expression in malignant breast together with the anti-proliferative and anti-invasive effects of the receptor upon upregulation in cancer cells represents the strongest indication of a tumor suppressor function.

In this review we first discuss what we have learned about the biology of ERβ from the complete and tissue-specific knockout mouse models. We also present information from studies looking at the role of the receptor in cell proliferation, invasion and metastasis. Since breast cancer is one of the most well-studied disease models for ERβ function, we additionally focus on the involvement of the receptor in the prevention and treatment of refractory disease. Considering the potential of targeting ERβ in breast cancer, we also discuss recent advancements in some of the most commonly used ERβ synthetic agonists. Finally, given the well-documented implication of tumor immunity on cancer progression, we explore the theme of estrogen receptor action in the tumor microenvironment.

## 2. The Phenotypes of ERβ Knockout Mouse Models

Several mouse models with constitutive deletion of ERβ in all tissues have been generated to date [16–19]. Earlier models were developed involving either deletion of exon 3 by the Cre-LoxP system [17,19] or disruption of the gene with the insertion of a neo cassette into exon 3 of the receptor in embryonic stem cells by homologous recombination [16]. The key finding that was consistent throughout the analysis of the three original knockout models was the severe disruption of ovulation in the absence of ERβ. The phenotyping of the latest mouse model with the crispr-cas9-mediated knockout of ERβ reports a tumor suppressor function in the ventral prostate and mammary epithelium, where the loss of the receptor leads to increased activity of the androgen receptor (AR) and ERα, respectively [18]. Although the expression of genes that are involved in the promotion of prostate cancer increased upon ERβ deletion, loss of the receptor alone did not lead to the development of prostate cancer [16]. Instead, ventral prostate epithelial hyperplasia and intraductal cancer-like lesions were observed in the absence of ERβ, which is consistent with the significantly higher number of ki67- and p63-positive cells in the same tissue of six-month-old ERβ knockout mice [18]. Of note, epithelial hyperplasia declined in the prostate of the same mice by the time they reached 18 months of age, linking this process to the physiology of the gland and the activity of the receptor in earlier stages of adult life [18].

Deletion of ERβ in mice was also shown to reduce the differentiation of mammary gland epithelium and decrease the levels of the adhesion molecules E-cadherin, connexin 32, occludin and integrin α [16]. The mammary gland of ERβ knockout mice had increased expression of ERα, PR and ki67 as well as more invasive epithelium with a higher expression of matrix metalloproteinases (MMPs) [18]. These results point to a key role for ERβ in controlling growth and promote the differentiation of prostate and mammary gland tissues in mice.

## 3. Effects of Tissue-Specific Deletion of ERβ

Several tissue-specific ERβ knockout mouse models have been developed and analyzed to characterize functions of the receptor on certain mouse organs. Muscle-specific deletion of ERβ led to a decrease in muscle mass and strength in female mice only [20]. Although ERβ is expressed in both male and female mice, genetic ablation of ERβ in muscle stem cells (satellite cells) caused impaired muscle regeneration following injury specifically in young female mice, pointing to the importance of ERβ in post-natal muscle growth [20]. On the other hand, deletion of ERβ in intestinal epithelial cells revealed a protective role of the receptor in colitis-induced adenomas by countering TNFα- and NFkB-mediated inflammation [21]. Knockout of ERβ induced more colorectal tumors in male mice, while female mice developed significantly larger tumors in the absence of the receptor [21]. Intestine-specific depletion of ERβ was also found to limit diversity in the gut microbiome during chemically induced colitis leading to colorectal cancer (CRC) [22]. In another study that was designed to test the selective activation of ERα or ERβ with specific ligands on the oncogenic activity of high fat diet (HFD), activation of ERβ elicited anti-inflammatory effects in the colon, as manifested by significantly reduced macrophage infiltration in both male and female mice [23], and protected against HFD-induced proliferation of colonic epithelial cells [23].

In addition to the intestine, effects of ERβ were investigated in mouse mammary gland using tissue-specific knockout models. Effective synergism between ERβ and the p53 tumor suppressor function was noted in breast cancer upon conditional deletion of both genes in mammary epithelial cells [24]. While knockout of ERβ alone did not give rise to mammary tumors, loss of the receptor in p53-defective tissues significantly shortened tumor latency compared with the conditional deletion of p53 alone [24]. Because ERβ has been reported to interact with both wild-type and mutant p53 enhancing the tumor suppressor function of the protein, the observed synergistic anti-tumor activity was linked to the function of the receptor in the developing mammary gland. During this period of extensive growth with an anticipated reduced capacity for genome surveillance due to p53 inactivation, loss of ERβ signaling can lead to the induction of aberrant cell proliferation and impaired differentiation and DNA repair to malignant transformation [25]

## 4. Post-Translational Modifications of ERβ

Serine residue (S) at positions 75, 87 and 105 in human ERβ were found to be targets of ERK1/2 and p38 kinases. S105 of endogenous ERβ was shown to undergo phosphorylation in MDA-MB-231 and BT-474 cells, enhancing the ability of the receptor to inhibit cell migration and invasion in vitro without affecting cell growth and cell cycle progression [26]. Consistent with the in vitro anti-tumor activity, phosphorylation of ERβ at S105 has been associated with a favorable prognosis in breast cancer [27]. On the other hand, the highly conserved MAPK target site S87 of ERβ was found to undergo phosphorylation by the stromal cell-derived growth factor 1 (SDF-1 or CXCL12) that enhances the occupancy of ERβ at EREs and AP1 sites, even in presence of tamoxifen [28].

Phosphorylation of ERβ at serine residues at positions 106 and 124 by MAP kinase has been shown to increase the interaction of ERβ with the co-activator SRC-1. When MAP kinase is activated by Ras, EGF or IGF-1, it stimulates the phosphorylation of serines in AF-1 of ERβ leading to an increased interaction with SRC-1 and ligand-independent activation of the receptor [29]. In addition to the human receptor, S16 in mouse ERβ was shown to be either phosphorylated or modified through O-glycosylation. Phosphorylation of S16 accelerated the degradation of mouse ERβ, whereas O-glycosylation of the same residue increased the stability of the receptor and its transcriptional activity [30,31].

Interestingly, the phosphorylation status of tyrosine 36 (Y36) of ERβ was found to be under the diametrically opposite control of c-ABL tyrosine kinase and EYA2 phosphatase. Phosphorylation of Y36 that is increased by the agonists 17β-estradiol, diarylpropionitrile (DPN) and S-equol is required for recruitment of ERβ co-activators to the promoters of target genes and causes inhibition of ERα-induced cancer cell growth in vitro and in xenografts [32,33]. In addition to regulating interaction with co-activators, the same phosphorylation has been reported to increase the turnover of the receptor by decreasing protein stability since mutant ERβ (Y36F), where tyrosine was replaced with phenylalanine, was more resistant to ubiquitin-mediated protein degradation [32]. Lastly, another residue that was also found to undergo phosphorylation is S6. This phosphorylation is necessary for sumoylation of ERβ at Lysine 4 that can be enhanced by constitutively active MAP/ERK kinases [34]. Sumoylation-deficient ERβ mutants displayed greater transcriptional activity in response to estrogen treatment, indicating the negative effect of sumoylation on the activity of the receptor, considering the function of Lysine-4 of ERβ as a suitable site for ubiquitination [34].

## 5. ERβ and Breast Cancer Cell Proliferation

Early studies using Taqman-based qPCR analysis showed that in contrast to ERα, which is upregulated in luminal tumors, ERβ mRNA levels were lower in breast tumor samples compared to normal breast tissue [11]. Consistent with the mRNA levels, quantitative immunohistochemistry clearly demonstrated higher expression of ERβ in benign breast with a sharp decline in breast carcinoma in situ (CIS) [35]. In line with the higher levels of ERβ in differentiated epithelium, the expression of the receptor exhibited a strong inverse correlation with that of the proliferation marker ki67 in ductal carcinoma in situ [35].

Despite the decrease of ERβ expression in malignant breast, a significant number of specimens from patients with hormone receptor-positive breast cancer were reported positive for ERβ. Expression of the receptor was strongly associated with pre-menopausal status and markers of less aggressive phenotypes, including axillary lymph node negativity and lower S phase fractions [36]. At the preclinical level, introducing ERβ in ERα-positive MCF-7 cells inhibited their proliferation in vitro and in xenografts in vivo. Expression of ERβ also caused G2 cell cycle arrest by repressing the transcription of cyclin D1, cyclin A and c-myc [37], and similar effects were observed in another luminal breast cancer cell line, the T47D cells [38]. ERβ was also found to counter Akt signaling by downregulating the upstream HER2/HER3 receptor dimer and upregulating the tumor suppressor PTEN that is known to inhibit Akt signaling in breast cancer cells [39].

ERβ can regulate the expression of oncogenes and tumor suppressors in luminal breast cancer cells in an ERα-dependent or independent fashion. ERα and ERβ can form heterodimers that control the recruitment of the co-activator SRC-1 [40–42]. ERα/ERβ heterodimers are less efficient than ERα homodimers in transactivating target genes, implying an inhibitory effect of ERβ in the transcriptional activity of ERα. In support of this mechanism of action, a microarray-based transcriptomic analysis in T47D cells revealed inhibition of ERα target gene expression upon induction of ERβ expression. Genes that were induced by ERα and repressed by ERβ were involved in cell proliferation, a finding that was also supported by in vitro cell proliferation assays [43]. Consistent with these studies and other previous findings [37], over-expression of ERβ in MCF-7 cells led to a marked decrease of estrogen-induced cell proliferation, and the analysis of binding sites in ERβ-transfected cells identified gene expression signatures that correlate with the inhibition of cell proliferation [44]. In agreement with the in vitro studies, less orthotopic ERβ-expressing T47D tumors were developed in SCID mice compared to xenografts that did not express the receptor [45]. Tumors expressing ERβ also had fewer blood vessels and reduced expression of proangiogenic factors [45]. The anti-proliferative effects of ERβ were primarily observed in ERα-positive breast cancer cells, suggesting that this specific function may result from interference with the pro-proliferative activity of ERα. This interaction in cells with variable expression of artificially introduced ERβ may also explain the publication of inconsistent data on cell proliferation in ERα-positive cells [46].

## 6. ERβ in Cancer Cell Invasion and Metastasis

In one of the earliest studies, the tumor repressive role of ERβ in prostate cancer was demonstrated through its adenovirus-driven expression in ERα-negative DU145 prostate cancer cells that caused a significant reduction of invasion in a matrigel-coated transwell assay [47]. Similar to DU145 cells, treatment of ERβ-transfected PC3 prostate cancer cells with agonists decreased cell migration and induced the expression of INPP4B, a repressor of Akt signaling [48]. In addition, overexpression of ERβ also reduced cell viability, migration, and inflammation and enhanced apoptosis in PC-3 and DU145 cells by suppressing lipopolysaccharide (LPS)-induced activation of NFkB that represents another driver of prostate cancer progression and mediator of inflammation [49].

Unlike the documented tumor suppressor role of ERβ in breast, prostate, ovarian, renal and thyroid cancer [50], reports of ERβ action in lung cancer have been controversial [51], with several groups supporting an oncogenic function of the receptor [52–58]. A recent study in non-small-cell lung cancer (NSCLC) cells showed that ERβ promotes invasion by directly binding to and inducing the expression of TMX4 circular RNA, which, through the inhibition of miR-622, leads to upregulation of the G protein-coupled receptor (GPCR) CXCR4 that promotes metastasis in several types of cancer [53]. Consistent with this function, knockdown of ERβ in lung cancer cells led to reduced vasculogenic mimicry and invasion, whereas overexpression of ERβ had the opposite effect [59]. ERβ also promoted cell invasion by directly binding to the regulatory region of lnc-RNA MALAT1 and increasing its expression, which, in turn, downregulates miR-145–5p and upregulates the oncogenic factor NEDD9 [59]. In line with the preclinical data, female patients with ERβ-positive NSCLC tumors had worse 5-year survival compared to those without ERβ expression [59]. Although an oncogenic function of ERβ in the lung should not be excluded and may be associated with the biology of the tissue, the pro-invasive effects in cancer cells following upregulation of the receptor need to be considered with extra caution and further validated to exclude non-physiological activation by the artificial expression.

**Figure 1.**
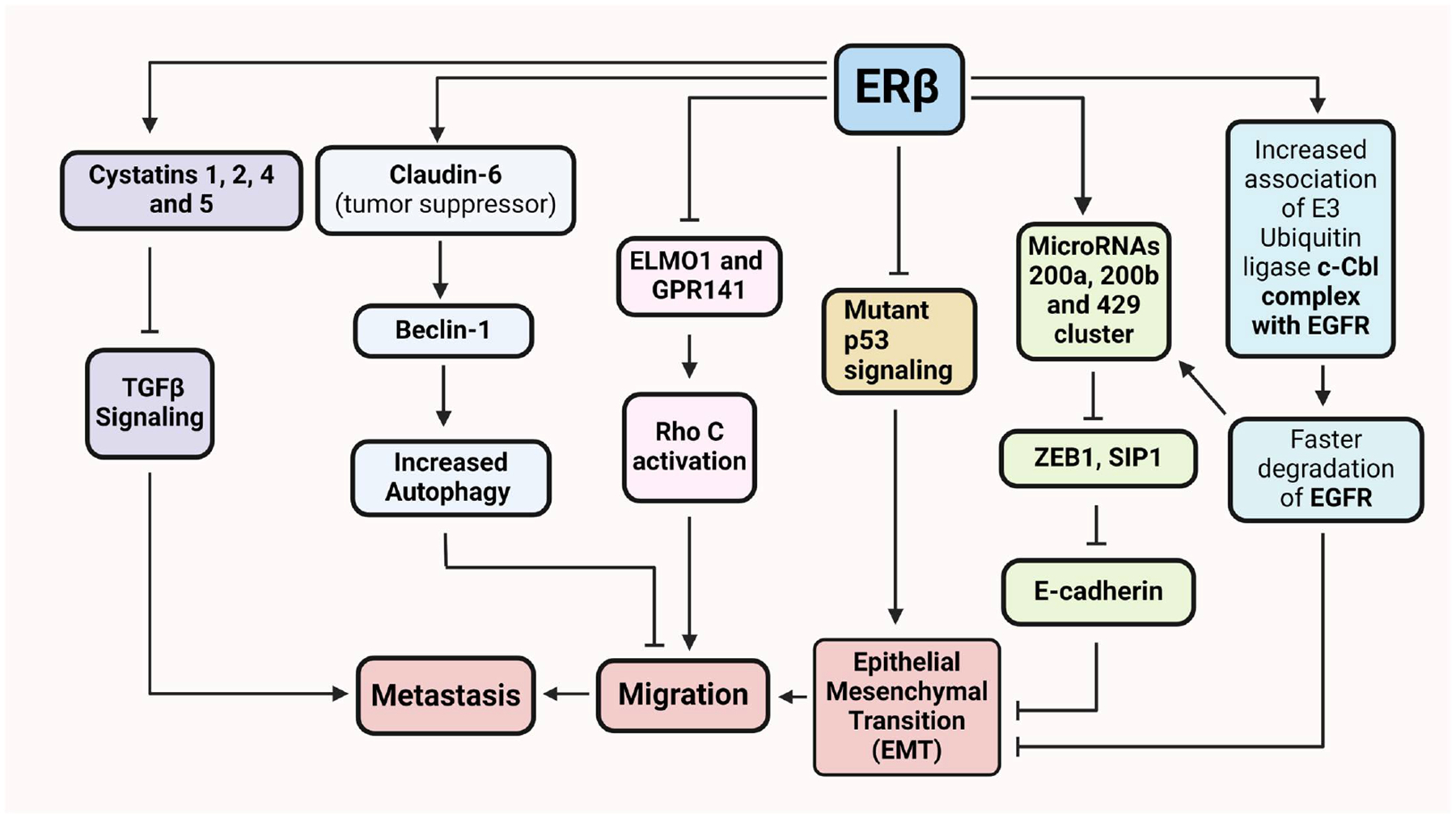
Flow chart depicting effects of ERβ on EMT, cell migration and metastasis in breast cancer. Cystatins 1, 2, 4 and 5 are direct targets of ERβ in triple negative breast cancer (TNBC) cells. Expression of ERβ in TNBC cells followed by agonist activation inhibits metastasis in vivo by inducing the expression of cystatins that downregulate TGFβ signaling. Beclin-1, a key regulator of autophagy is upregulated by Claudin-6, a direct target of ERβ in breast cancer. Claudin-6 inhibits breast cancer cell migration and invasion. ERβ represses transcription of the activators of the cytoskeleton remodeler RhoC, ELMO1 and GRP141, by directly binding to their regulatory regions, thereby preventing RhoC activation and actin-based cell migration. Approximately 80% of TNBCs harbor oncogenic mutations of p53. ERβ directly interacts with mutant p53 and inhibits its prometastatic signaling. ERβ also inhibits epithelial–mesenchymal transition (EMT) by inducing EGFR degradation that results in upregulation of the epithelial markers miR-200a-b-429 and E-cadherin.

In contrast to lung cancer, there is consensus regarding the anti-invasive and anti-metastatic role of ERβ in breast cancer. Claudin-6 (CLDN6), a tight junction protein and tumor suppressor, was found to be a direct target of ERβ in breast cancer cells [60]. Treatment of MDA-MB-231 and ERβ-overexpressing SK-BR-3 breast cancer cells with the Erβ-specific agonist DPN caused autophagy through CLDN6-mediated upregulation of the key mediator of autophagy beclin-1 (Figure 1). Overexpression of CLDN6 in MDA-MB-231 cells also led to reduced lung and liver metastasis in mice [60]. Upregulation of ERβ in TNBC (triple negative breast cancer) MDA-MB-231 and Hs578T cell lines induced the expression of the epithelial marker E-cadherin and suppressed cell migration and invasion in vitro as well as in zebrafish embryos [61]. ERβ was found to promote ubiquitination and degradation of EGFR along with induction of members of the miR200 family leading to subsequent inhibition of the transcriptional repressors of E-cadherin SIP1 and ZEB1 (Figure 1). Consistent with the association in cancer cells, ERβ protein levels positively correlated with those of E-cadherin in clinical patient samples [61]. ERβ was also found to transcriptionally repress EGFR, thereby indirectly downregulating IMP3 to counter invasion and migration in TNBC [62]. G protein-coupled estrogen receptor 1 (GPER) is a membrane-bound estrogen receptor that has been shown, like other GPCRs, to transactivate EGFR [63]. GPER expression positively correlates with disease progression in breast cancer patients [64]. Since ERβ has already been shown to transcriptionally downregulate another G protein-coupled receptor—GPR141—to inhibit actin-based migration in inflammatory breast cancer [65], there is a possibility of functional crosstalk between ERβ and GPER or other GPCRs, where ERβ might oppose EGFR signaling by inhibiting the expression and activity of GPER. In addition to inhibiting EGFR, ERβ was shown to reduce invasion in TNBC cells by directly interacting with and blocking transcription by the oncogenic mutant p53 that exists in about 80% of TNBCs (Figure 1) [25]. Similarly, ligand-mediated activation of ERβ in TNBC cells resulted in their decreased invasion and in vivo lung colonization through upregulation of several members of the family of cystatins via direct binding of the receptor to their regulatory elements (Figure 1) [66]. The importance of cystatins for TNBC metastasis was verified by their ability to decrease the invasiveness of TNBC cells by repressing TGFβ signaling and their association with longer recurrence-free survival (RFS) in patients (Figure 1) [66]. In addition to TNBC, ERβ was found to decrease the invasiveness of inflammatory breast cancer (IBC) cells by downregulating GPR141 and the guanine nucleotide exchange factor (GEF)-interacting protein ELMO1 that activate the mediator of IBC metastasis RhoC [65]. In contrast to ERβ, a few studies provided conflicting evidence about the role of ERα in the invasiveness and metastatic potential of breast cancer cells. Initially, silencing of ERα has been shown to cause epithelial to mesenchymal transition (EMT) in ERα-positive breast cancer cells [67]. Subsequently, ERα has been reported to promote breast cancer cell migration and invasion by actin cytoskeletal remodeling through focal adhesion kinase (FAK) and N-WASP [68] and through Rho-associated kinase 2 (ROCK-2) [69].

## 7. ERβ in TNBC

TNBC is marked by the absence of the receptors ERα, PR and HER2 that have been validated as oncogenic drivers in other subtypes of breast cancer. Although TNBC accounts for approximately 15% of total breast cancers, it is responsible for the majority of breast cancer-associated deaths [70]. This is, in part, due to the high propensity of TNBC tumors to develop metastasis, the high frequency of resistance to standard chemotherapy and the lack of effective targeted therapy.

**Figure 2.**
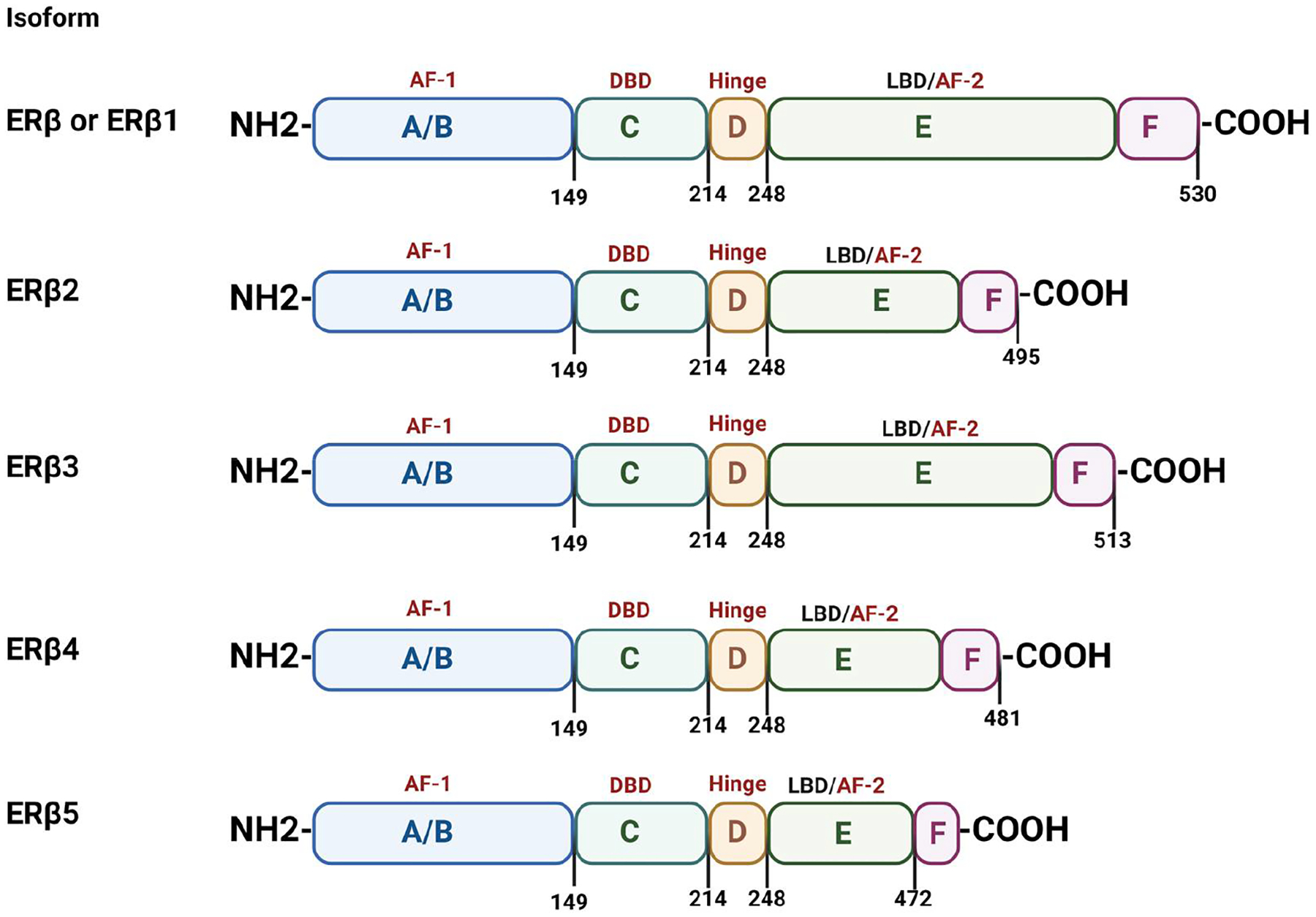
Structural and functional domains of estrogen receptor β. Domains are shown for both the full length ERβ (also called ERβ1) and its isoforms ERβ2–5. Numbers indicate amino acid length of individual domains and the full length proteins. All ERβ isoforms are identical until the hinge region, where they begin diverging from the C-terminal of the ligand-binding domain (LBD). The ligand-independent transactivation function (AF-1) resides in the N-terminus of the receptor and serves as an interaction site for regulatory factors. The DNA-binding domain (DBD) recognizes estrogen response elements (ERE) in regulatory regions of target genes, whereas the hinge region harbors a nuclear localization signal (NLS). The LBD consists of the ligand-binding transactivation function (AF-2) and provides an interface for receptor dimerization and co-activator binding.

Different isoforms of ERβ have been associated with clinical outcomes in TNBC. These include the full length ERβ (also known as ERβ1) that is composed of 530 amino acids and is the only isoform that forms homodimers and heterodimers and bind ligands [71]. On the other hand, the variants ERβ2, 3, 4 and 5 that result from alternative splicing of the last coding exon form heterodimers with ERβ1 and have an impaired ability to bind ligands (Figure 2) [71]. The expression of ERβ1 has been reported in about 18–27% of TNBC cases, and earlier studies indicated the importance of the receptor as an independent predictor of a favorable prognosis [72–76]. As per a recent report from Katzenellenbogen lab, ERβ2 and ERβ5 are the most abundant isoforms in TNBC cell lines and tumors, whereas ERβ1 is barely detectable. In contrast to ERβ1, the variants 2 and 5 were found to elicit an oncogenic function since knockdown in TNBC cells decreased proliferation, migration and invasion, whereas their overexpression had the opposite effect on these specific cellular phenotypes [71,77]. At the mechanistic level, overexpression of ERβ1 and treatment with specific ligands increased the protein levels of the epithelial and anti-invasive marker E-cadherin [61,62]. Upregulation of ERβ1 also reduced the expression of the oncogenic survivin (BIRC5), similar to the depletion of ERβ2 and ERβ5, suggesting opposing effects of ERβ isoforms on gene regulation [77]. The oncogenic activity of the variants is in agreement with previous findings, suggesting an association of ERβ2 with poor prognosis in hormone receptor-negative breast cancer [73,77]. Similar to TNBC, in high-grade serous ovarian cancer, where more than 95% of the tumors harbor p53 mutations, ERβ2 was found to partner with mutant p53 to increase the transcription of FOXM1, leading to enhanced proliferation and therapy resistance [78]. The transcriptional activation of mutant p53 by ERβ2 represents another example of the opposite function of the variants in cancer considering the previously reported inhibitory interaction of ERβ1 with mutant p53 in TNBC [25].

In addition to mutant p53, the full length ERβ has been shown to inhibit the function of other known drivers of TNBC. Expression of ERβ1 has been reported to induce apoptosis and reduce the proliferation and metastatic potential of androgen receptor (AR)-positive TNBC cells [76]. Similar, upregulation of ERβ1 increased the sensitivity of the same cells to the AR inhibitor enzalutamide [79]. ERβ1 decreased the activity of AR by forming heterodimers and inhibiting PI3K/Akt signaling. The same variant was also shown to inhibit proliferation and migration of TNBC cells by forming a co-repressor complex with PRC2/EZH2 to repress the transcription of p65/RelA and downregulate the NFkB pathway [75,76]. Similar to upregulation, treatment with the ERβ agonist liquiritigenin inhibited cell proliferation and increased the sensitivity of TNBC cells to doxorubicin [80]. As with the cell proliferation, treatment with ERβ agonists greatly mitigated the invasion of TNBC cells when they were grown alone [81] and during their co-culture with MG63 osteoblasts [82]. Lastly, ERβ1 was also found in the same cells to downregulate the oncogenic pathway of cholesterol biosynthesis by binding to the promoter of SREBP1 [76]. The opposing effects of the variants together with their variable expression in tumors may have accounted for the initial controversy surrounding the role of ERβ in TNBC [83,84]. Recent findings, however, have improved our understanding about the exact actions of the receptor and its clinical importance for the disease [85,86].

## 8. Synthetic Ligands and ERβ Activity

The nature of the estrogenic ligand effect largely depends on the conformation of the ligand-binding domain (LBD) of ER subtypes. ERα and ERβ share a medium homology of 59% in the primary protein sequence of LBD [87]. The LBD in both isoforms has important features such as the ligand-dependent transcription activation function (AF2), a homo- or hetero-dimerization interface and an interaction surface for co-regulators [88]. The LBDs of ERα and ERβ have a similar globular structure and consist of 11 α-helices organized as a three-layered sandwich structure with helices 4, 5, 6, 8 and 9 flanked on one side by helices 1 and 3, and by helices 7, 10 and 11 on the other [88,89]. 22 hydrophobic residues line the ligand-binding cavity in ERs and interact with the ligand [90]. The orientation of helix 12 with respect to the ligand-binding pocket determines whether a ligand serves as an agonist or antagonist. In an agonist-bound conformation, helix 12 is positioned at the entrance of a ligand-binding cavity and serves as an interaction surface for nuclear receptor co-activators [91]. Antagonists alter helix 12 positioning in a manner that blocks recruitment of co-activators [91]. The ligand-binding cavity of ERβ is smaller in size and narrower compared to that of ERα. ERβ also differs from ERα in two residues out of the 22 that form the ligand-binding cavity. Leu384 and Met421 in ERα are replaced by Met336 and Ile373, respectively, in ERβ [87,89].

### 8.1. Raloxifene

**Figure 3.**
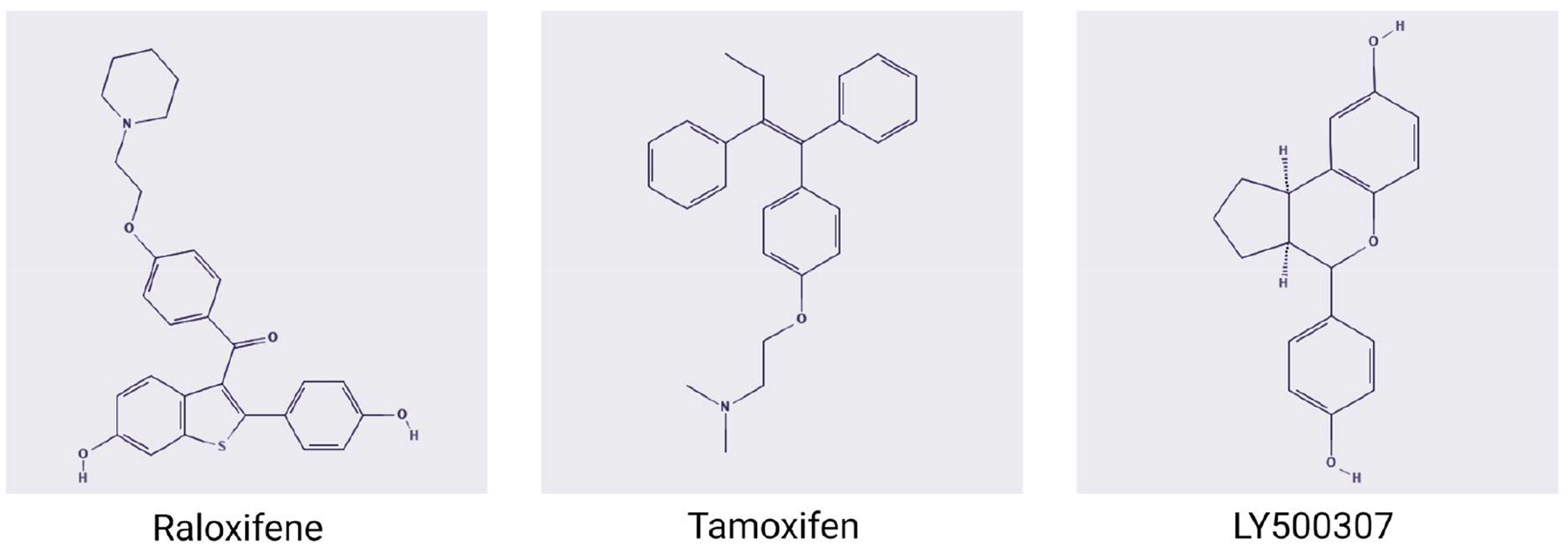
Chemical structure of synthetic ligands of estrogen receptors tamoxifen, raloxifene and LY500307.

Raloxifene binds both ERα and ERβ with high affinity (Figure 3) [92]. It acts as an ERα antagonist in the mammary gland and uterus and as an agonist in bone and the liver [93]. In a clinical trial with more than 10,000 post-menopausal women, raloxifene significantly reduced the risk of invasive breast cancer, had no effects on outcomes that were associated with coronary heart disease, but increased the risk of fatal stroke [94] and venous thromboembolism [94,95]. This ER ligand was also found to increase bone mineral density and lower the levels of total cholesterol and LDL in post-menopausal women [96] and, hence, it was approved for the treatment of osteoporosis in post-menopausal women in 2007. In addition to ERα, recent preclinical studies showed that the nano formulation of raloxifene inhibited TNBC tumor growth in vitro and in vivo, partially through regulating the activity of ERβ [97]. Similar to TNBC, the drug was also shown to inhibit migration of hepatocellular cancer cells through ERβ-mediated inhibition of the Akt signaling pathway [98]. Raloxifene was further found to inhibit the progression of pancreatic ductal adenocarcinoma (PDAC) in an orthotopic xenograft model through ERβ [99]. Moreover, it mitigated metastasis and elicited tumor suppressive effects in AR-negative and castration-resistant prostate cancer (CRPC) [100]. These findings suggest a role for ERβ in repurposing ER ligands for use in treatment of ERα-negative malignancies.

### 8.2. Tamoxifen

Tamoxifen acts as an ERα antagonist in breast tissue and an agonist in the uterus, bone, and the liver (Figure 3) [101,102]. Tamoxifen was first approved by the FDA for the treatment of ERα-positive breast cancer in 1977 and later as an adjuvant treatment for primary breast cancer [103]. Application of tamoxifen therapy significantly benefited patients with the disease since it reduced breast cancer-associated mortality by a third [104]. Although ERα appears as the primary mediator of the clinical effects of the drug, new evidence suggests that tamoxifen can also affect breast cancer through ERβ. Initially, low ERβ protein levels in ERα-positive tumors were reported to predict resistance to tamoxifen [105,106], and treatment of breast cancer cells with ERβ-selective agonists enhanced the growth-inhibitory effects of tamoxifen [107–109]. Similarly, ERβ sensitized tamoxifen-resistant MCF-7 cells to endoplasmic reticulum (ER) stress apoptosis by downregulating the unfolded protein response (UPR) [109]. Tamoxifen was also shown to inhibit mutant p53-dependent oncogenic gene expression in ERβ-expressing but not in control MDA-MB-231 TNBC cells [25]. On the other hand, the ligand was found to engage mitochondrial ERβ, both as agonist and antagonist, thereby modulating the levels of manganese superoxide dismutase (MnSoD) and contributing to tamoxifen resistance [110]. In addition to breast cancer, targeting ERβ with tamoxifen in diffuse large B-cell lymphoma (DLBCL) reduced cell viability in vitro, an effect that was significantly mitigated with knockdown of ERβ [111] and corroborated in a xenograft lymphoma model [111].

### 8.3. LY500307

**Figure 4.**
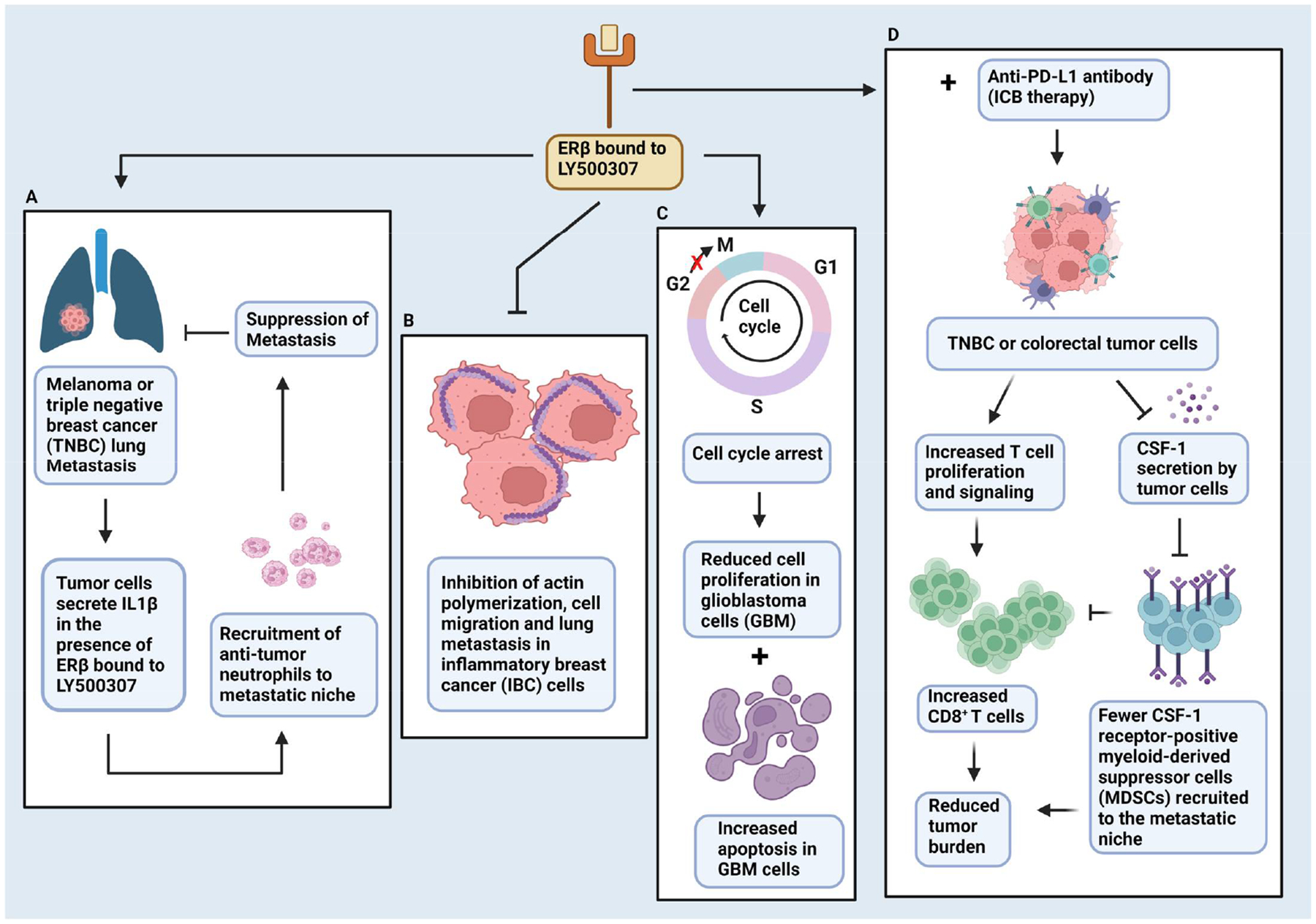
Anti-tumor activities of the selective ERβ agonist LY500307. (**A**) Activating ERβ with LY500307 enables triple negative breast cancer (TNBC) and melanoma cells to secrete interleukin-1β (IL-1β), which stimulates the recruitment of anti-tumor neutrophils to the metastatic niche suppressing lung metastasis. (**B**) Similar to TNBC, ERβ and LY500307 prevent lung metastasis in inflammatory breast cancer (IBC) by inhibiting actin-based cell migration through the repression of the direct targets GPR141 and ELMO1 that activate the cytoskeleton remodeler RhoC. (**C**) Activation of ERβ with LY500307 also inhibits tumor growth and increases the survival of mice with highly aggressive glioblastoma (GBM) by reducing cell proliferation and inducing cell cycle arrest and apoptosis. (**D**) LY500307 greatly improves the therapeutic efficacy of immune checkpoint blockade (ICB) therapy with anti-PDL1 antibodies in TNBC and colorectal cancer. Activating ERβ in tumor cells with LY500307 prevents them from secreting CSF-1 in the tumor microenvironment, thus diminishing the recruitment of myeloid-derived suppressor cells (MDSCs), which, along with increased CD8^+^ T cells, leads to smaller tumors in mice. → and ⊥ represent positive and negative regulation, respectively and Red X indicates blockade.

Several studies have reported the anti-tumor activity of the ERβ-selective agonist LY500307 since its development by researchers at Eli Lilly in 2012 (Figure 3). Treatment with LY500307 caused suppression of TNBC and melanoma lung colonization by inducing recruitment of neutrophils to the metastatic site (Figure 4A) [112]. The recruitment of neutrophils was associated with the expression and secretion of IL-1β from cancer cells, and the involvement of this specific cytokine was verified by the absence of anti-metastatic effects of LY500307 in IL-1β knockout mice [112]. Treatment with LY500307 also improved the efficacy of the PD-1 antibody in in vivo models of TNBC and colorectal cancer [113]. In addition to regulating neutrophils, LY500307 reduced the recruitment of CSF-1 receptor-positive myeloid-derived suppressor cells (MDSC) to the tumor microenvironment while also increasing CD8^+^ cytotoxic T cells by decreasing the production of CSF-1 by tumor cells (Figure 4D) [113]. Beyond TNBC, activating tumor-endogenous ERβ by treating mice bearing orthotopically implanted inflammatory breast cancer tumors with LY500307 led to reduced lung metastasis (Figure 4B) [65]. In addition to IBC, ERβ was also found to be enriched in ovarian cancer stem cells (OVSC), and treatment with LY500307 reduced their stemness and induced apoptosis [114]. Treatment with LY500307 also significantly impaired the tumor-initiating potential of OVSCs in orthotopically implanted xenograft models [114]. Similarly, glioblastomas (GBM) express ERβ, and treatment of GBM cells with this selective agonist reduced proliferation and enhanced apoptosis in vitro (Figure 4C). More importantly, the ligand improved the survival of GBM tumor-bearing mice and inhibited in vivo tumor growth [115]. In an attempt to potentiate ERβ signaling after treatment with LY500307 and driven by the observed increased acetylation of the ERβ promoter, glioblastoma cells were incubated with HDAC inhibitors. HDAC inhibitors did indeed increase the expression of ERβ and upregulated its target genes, and the combination of these inhibitors with LY500307 enhanced the survival of mice with orthotopic GBM tumors [116]. Similar results were observed in melanoma cell lines, where treatment with LY500307 reduced cell proliferation, increased apoptosis, decreased cell migration and partially reversed EMT [117]. Despite the enthusiasm generated as a result of the use of the compound in preclinical cancer models and its demonstrated in vivo anti-tumor activity, LY500307 failed to show clinical efficacy in clinical trials for other conditions, including men with benign prostatic hyperplasia (BPH) [118] and patients with cognitive impairment associated with schizophrenia [119].

## 9. ERβ and the Tumor Microenvironment

Despite the documented association of estrogen signaling with the function of the immune system in various pathophysiological conditions, the role of estrogen receptors in regulating tumor immunity still remains elusive, partially due to the lack of appropriate syngeneic and transgenic immunocompetent tumor models. Only a few studies have explored the function of ERβ in the tumor microenvironment and how this impacts cancer progression and metastasis. One of these studies has focused on bladder cancer, where ERβ has been detected as the predominant ER subtype in both cell lines and tumor samples [120]. Higher expression of ERβ has been reported in metastatic tissue compared to benign urothelium and is strongly correlated with more aggressive phenotypes [121]. This correlation has been explained by the recruitment of ERβ-expressing mast cells [122] and CD4^+^ T cells [123] in bladder cancer and by the association of mast and CD4^+^ T cells in the tumor microenvironment with enhanced invasiveness of bladder cancer cells. The effect of ERβ was verified by treatment with the ERβ antagonist PHTPP or ERβ shRNA that abrogated the increased invasiveness of bladder cancer cells [122].

In addition to bladder cancer, estrogen signaling has been linked to the microenvironment of breast cancer. However, most of the evidence supporting this association has been derived from the study of ERα. It is well accepted that immune cell infiltrates in the breast tumor microenvironment are altered based on ERα status [124]. For example, numerous studies have shown an association between high eosinophil count in peripheral blood and survival benefit in patients with ERα-negative breast cancer [125–127]. On the other hand, ERα is known to mediate the immune-suppressive effects of 17β-estradiol that are, in part, dependent on FoxP3-positive regulatory T cells (Tregs) [128]. In addition to recruitment, treatment with 17β-estradiol increased the activity of Tregs by inducing their intracellular expression of PD-1. The involvement of ERα was confirmed through the analysis of ERα knockout mice where the intracellular expression of PD-1 and Treg-mediated immune suppression were reduced [129].

The involvement of Tregs in estrogen-associated immunoregulation has also been observed in other conditions. These include the auto-immune disorders such as inflammatory bowel disease (IBD), where ERβ as the predominant ER subtype in intestinal mucosa [130] was shown to elicit potent anti-inflammatory effects [130–134]. The population of ERβ^+^CD4^+^ T cells was significantly lower in experimentally induced IBD and was associated with increased disease severity [135,136]. Treatment with ERβ-specific agonists countered inflammation in IBD by inducing differentiation of naïve T cells to Tregs and inhibiting pro-inflammatory T cell responses [135].

In addition to Tregs, ERβ has been shown to affect breast cancer by regulating other components of the immune system. The selective ERβ agonist LY500307 has been reported to suppress metastasis of TNBC by inducing tumor cell expression and secretion of IL-1β, resulting in subsequent recruitment of neutrophils to the metastatic site [112]. The same ligand also increased the sensitivity of TNBC tumors to PD-1-based immunotherapy [113]. In addition to neutrophils, LY500307 inhibited the expression of CSF-1 in tumor cells, leading to reduced recruitment of myeloid-derived suppressor cells (MDSC) and an increase in CD8^+^ cytotoxic T cells in the tumor microenvironment [113]. The role of ERβ signaling in the cells of the tumor microenvironment was evaluated by generating mice with a mutated mouse ERβ, where the tyrosine 55 residue that is equivalent to Y36 in human ERβ and essential for maintaining an active receptor through phosphorylation was replaced with a phenylalanine. Mice with whole body homozygous mutated ERβ unable to undergo phosphorylation exhibited significantly faster growth of orthotopically implanted syngeneic mammary and melanoma tumors [137]. Replacing bone marrow of wild-type mice with bone marrow from ERβ mutant mice led to fewer tumor-infiltrating CD8^+^ and CD4^+^ T cells compared to the control mice, indicating the impaired ability of host immune cells to control tumor growth in the absence of ERβ signaling. Indeed, CD8^+^ T cells in mice with a mutated ERβ phosphotyrosine switch (Y55) produced lower amounts of anti-tumor cytokines [137].

## 10. Concluding Remarks

Although initial reports of ERβ action were conflicting, generating skepticism about the role and clinical importance of the receptor, extensive research in the last decade has increased our confidence for a tumor suppressor function in breast cancer. In addition to the direct effects of the receptor on tumor cells, recent studies have revealed entirely new courses of action through regulation of the tumor microenvironment. Activation of ERβ in tumor cells is now known to alter tumor immunity through the secretion of immunomodulatory cytokines [112,113]. In addition to tumor epithelial cells, ERβ is expressed in various cell types including endothelial cells, fibroblasts and tumor-infiltrating lymphocytes [138–141]. This underscores the importance of determining the ability of ERβ to signal from the tumor microenvironment to regulate tumor development and metastasis and the capacity of ligands to enhance ERβ signaling in these cells and achieve favorable clinical outcomes by potentiating the anti-tumor activity of the host immune system. As ERβ has been reported to act as a tumor suppressor in various malignancies including breast, prostate, colorectal, ovarian and glioblastoma [50], further research to corroborate this function and define the mechanism of action will be essential in order to determine the value of the receptor as biomarker and therapeutic target and its utility in new approaches to combat the resistant and metastatic states of these diseases.

## References

[1] Jensen E Studies of growth phenomena using tritium labeled steroids. In Proceedings of the 4th International Congress of Biochemistry, Vienna, Austria, 1–6 September 1958; Pergamon Press: London, UK; p. 119.

[2] Kuiper GG; Enmark E; Pelto-Huikko M; Nilsson S; Gustafsson JA Cloning of a novel receptor expressed in rat prostate and ovary. Proc. Natl. Acad. Sci. USA 1996, 93, 5925–5930. 8650195 10.1073/pnas.93.12.5925 PMC39164

[3] Enmark E; Pelto-Huikko M; Grandien K; Lagercrantz S; Lagercrantz J; Fried G; Nordenskjold M; Gustafsson JA Human estrogen receptor beta-gene structure, chromosomal localization, and expression pattern. J. Clin. Endocrinol. Metab 1997, 82, 4258–4265. 9398750 10.1210/jcem.82.12.4470

[4] Mosselman S; Polman J; Dijkema R ERβ: Identification and characterization of a novel human estrogen receptor. FEBS Lett. 1996, 392, 49–53. 8769313 10.1016/0014-5793(96)00782-x

[5] Kuiper GG; Shughrue PJ; Merchenthaler I; Gustafsson JA The estrogen receptor beta subtype: A novel mediator of estrogen action in neuroendocrine systems. Front. Neuroendocrinol 1998, 19, 253–286. 9799586 10.1006/frne.1998.0170

[6] Taylor AH; Al-Azzawi F Immunolocalisation of oestrogen receptor beta in human tissues. J. Mol. Endocrinol 2000, 24, 145–155. 10657006 10.1677/jme.0.0240145

[7] Drummond AE; Fuller P The importance of ERβ signalling in the ovary. J. Endocrinol 2010, 205, 15–23. 20019181 10.1677/JOE-09-0379

[8] Jia M; Dahlman-Wright K; Gustafsson JA Estrogen receptor alpha and beta in health and disease. Best Pract. Res. Clin. Endocrinol. Metab 2015, 29, 557–568. 26303083 10.1016/j.beem.2015.04.008

[9] Siegel RL; Miller KD; Wagle NS; Jemal A Cancer statistics, 2023. Ca Cancer J. Clin 2023, 73, 17–48. 36633525 10.3322/caac.21763

[10] Speirs V; Parkes AT; Kerin MJ; Walton DS; Carleton PJ; Fox JN; Atkin SL Coexpression of estrogen receptor α and β: Poor prognostic factors in human breast cancer? Cancer Res. 1999, 59, 525–528. 9973193

[11] Iwao K; Miyoshi Y; Egawa C; Ikeda N; Noguchi S Quantitative analysis of estrogen receptor-β mRNA and its variants in human breast cancers. Int. J. Cancer 2000, 88, 733–736. 11072241 10.1002/1097-0215(20001201)88:5<733::aid-ijc8>3.0.co;2-m

[12] Shaw JA; Udokang K; Mosquera JM; Chauhan H; Jones JL; Walker RA Oestrogen receptors alpha and beta differ in normal human breast and breast carcinomas. J. Pathol. A J. Pathol. Soc. Great Br. Irel 2002, 198, 450–457. 10.1002/path.1230 12434414

[13] Park B-W; Kim K-S; Heo M-K; Ko S-S; Lee KS; Hong SW; Yang W-I; Kim J-H; Kim GE treatment. Expression of estrogen receptor-β in normal mammary and tumor tissues: Is it protective in breast carcinogenesis? Breast Cancer Res. Treat 2003, 80, 79–85. 12889601 10.1023/A:1024406223619

[14] Skliris GP; Munot K; Bell SM; Carder PJ; Lane S; Horgan K; Lansdown MR; Parkes AT; Hanby AM; Markham AF; Reduced expression of oestrogen receptor β in invasive breast cancer and its re-expression using DNA methyl transferase inhibitors in a cell line model. J. Pathol. A J. Pathol. Soc. Great Br. Irel 2003, 201, 213–220. 10.1002/path.1436 14517838

[15] Rody A; Holtrich U; Solbach C; Kourtis K; Von Minckwitz G; Engels K; Kissler S; Gatje R; Karn T; Kaufmann M Methylation of estrogen receptor β promoter correlates with loss of ER-β expression in mammary carcinoma and is an early indication marker in premalignant lesions. Endocr.-Relat. Cancer 2005, 12, 903–916. 16322330 10.1677/erc.1.01088

[16] Krege JH; Hodgin JB; Couse JF; Enmark E; Warner M; Mahler JF; Sar M; Korach KS; Gustafsson J-Å; Smithies O Generation and reproductive phenotypes of mice lacking estrogen receptor β. Proc. Natl. Acad. Sci. USA 1998, 95, 15677–15682. 9861029 10.1073/pnas.95.26.15677 PMC28103

[17] Antal MC; Krust A; Chambon P; Mark M Sterility and absence of histopathological defects in nonreproductive organs of a mouse ERβ-null mutant. Proc. Natl. Acad. Sci. USA 2008, 105, 2433–2438. 18268329 10.1073/pnas.0712029105 PMC2268154

[18] Warner M; Wu W.-f.; Montanholi L; Nalvarte I; Antonson P; Gustafsson J-A Ventral prostate and mammary gland phenotype in mice with complete deletion of the ERβ gene. Proc. Natl. Acad. Sci. USA 2020, 117, 4902–4909. 32075916 10.1073/pnas.1920478117 PMC7060692

[19] Dupont S; Krust A; Gansmuller A; Dierich A; Chambon P; Mark M Effect of single and compound knockouts of estrogen receptors α (ERα) and β (ERβ) on mouse reproductive phenotypes. Development 2000, 127, 4277–4291. 10976058 10.1242/dev.127.19.4277

[20] Seko D; Fujita R; Kitajima Y; Nakamura K; Imai Y; Ono Y Estrogen receptor β controls muscle growth and regeneration in young female mice. Stem Cell Rep. 2020, 15, 577–586. 10.1016/j.stemcr.2020.07.017 PMC7486216 32822588

[21] Hases L; Indukuri R; Birgersson M; Nguyen-Vu T; Lozano R; Saxena A; Hartman J; Frasor J; Gustafsson J-Å; Katajisto P Intestinal estrogen receptor beta suppresses colon inflammation and tumorigenesis in both sexes. Cancer Lett. 2020, 492, 54–62. 32711097 10.1016/j.canlet.2020.06.021

[22] Ibrahim A; Hugerth LW; Hases L; Saxena A; Seifert M; Thomas Q; Gustafsson JÅ; Engstrand L; Williams C Colitisinduced colorectal cancer and intestinal epithelial estrogen receptor beta impact gut microbiota diversity. Int. J. Cancer 2019, 144, 3086–3098. 30515752 10.1002/ijc.32037 PMC6519213

[23] Hases L; Archer A; Indukuri R; Birgersson M; Savva C; Korach-André M; Williams C High-fat diet and estrogen impacts the colon and its transcriptome in a sex-dependent manner. Sci. Rep 2020, 10, 16160. 32999402 10.1038/s41598-020-73166-1 PMC7527340

[24] Bado I; Nikolos F; Rajapaksa G; Wu W; Castaneda J; Krishnamurthy S; Webb P; Gustafsson J-Å; Thomas C Somatic loss of estrogen receptor beta and p53 synergize to induce breast tumorigenesis. Breast Cancer Res. 2017, 19, 79. 28673316 10.1186/s13058-017-0872-z PMC5494907

[25] Bado I; Nikolos F; Rajapaksa G; Gustafsson JA; Thomas C ERbeta decreases the invasiveness of triple-negative breast cancer cells by regulating mutant p53 oncogenic function. Oncotarget 2016, 7, 13599–13611. 26871946 10.18632/oncotarget.7300 PMC4924664

[26] Lam H-M; Babu CS; Wang J; Yuan Y; Lam Y-W; Ho S-M; Leung Y-K Phosphorylation of human estrogen receptor-beta at serine 105 inhibits breast cancer cell migration and invasion. Mol. Cell. Endocrinol 2012, 358, 27–35. 22370157 10.1016/j.mce.2012.02.012 PMC3348253

[27] Hamilton-Burke W; Coleman L; Cummings M; Green CA; Holliday DL; Horgan K; Maraqa L; Peter MB; Pollock S; Shaaban AM Phosphorylation of estrogen receptor β at serine 105 is associated with good prognosis in breast cancer. Am. J. Pathol 2010, 177, 1079–1086. 20696772 10.2353/ajpath.2010.090886 PMC2928942

[28] Sauvé K; Lepage J; Sanchez M; Heveker N; Tremblay A Positive feedback activation of estrogen receptors by the CXCL12-CXCR4 pathway. Cancer Res. 2009, 69, 5793–5800. 19584281 10.1158/0008-5472.CAN-08-4924

[29] Tremblay A; Tremblay GB; Labrie F; Giguère V Ligand-independent recruitment of SRC-1 to estrogen receptor β through phosphorylation of activation function AF-1. Mol. Cell 1999, 3, 513–519. 10230404 10.1016/s1097-2765(00)80479-7

[30] Cheng X; Hart GW AlternativeO-Glycosylation/O-Phosphorylation of Serine-16 in Murine Estrogen Receptor β: POST-TRANSLATIONAL REGULATION OF TURNOVER AND TRANSACTIVATION ACTIVITY. J. Biol. Chem 2001, 276, 10570–10575. 11150304 10.1074/jbc.M010411200

[31] Cheng X; Cole RN; Zaia J; Hart GW Alternative O-glycosylation/O-phosphorylation of the murine estrogen receptor β. Biochemistry 2000, 39, 11609–11620. 10995228 10.1021/bi000755i

[32] Yuan B; Cheng L; Gupta K; Chiang H-C; Gupta HB; Sareddy GR; Wang D; Lathrop K; Elledge R; Wang P Tyrosine phosphorylation regulates ERβ ubiquitination, protein turnover, and inhibition of breast cancer. Oncotarget 2016, 7, 42585. 27323858 10.18632/oncotarget.10018 PMC5173158

[33] Yuan B; Cheng L; Chiang HC; Xu X; Han Y; Su H; Wang L; Zhang B; Lin J; Li X; A phosphotyrosine switch determines the antitumor activity of ERbeta. J. Clin. Investig 2014, 124, 3378–3390. 24960160 10.1172/JCI74085 PMC4109526

[34] Picard N; Caron V; Bilodeau S; Sanchez M; Mascle X; Aubry M; Tremblay A Identification of estrogen receptor β as a SUMO-1 target reveals a novel phosphorylated sumoylation motif and regulation by glycogen synthase kinase 3β. Mol. Cell. Biol 2012, 32, 2709–2721. 22586270 10.1128/MCB.06624-11 PMC3416183

[35] Roger P; Sahla ME; Mäkelä S; Gustafsson J.A.k.; Baldet P; Rochefort H Decreased expression of estrogen receptor β protein in proliferative preinvasive mammary tumors. Cancer Res. 2001, 61, 2537–2541. 11289127

[36] Järvinen TA; Pelto-Huikko M; Holli K; Isola J Estrogen receptor β is coexpressed with ERα and PR and associated with nodal status, grade, and proliferation rate in breast cancer. Am. J. Pathol 2000, 156, 29–35. 10623650 10.1016/s0002-9440(10)64702-5 PMC1868617

[37] Paruthiyil S; Parmar H; Kerekatte V; Cunha GR; Firestone GL; Leitman DC Estrogen receptor β inhibits human breast cancer cell proliferation and tumor formation by causing a G2 cell cycle arrest. Cancer Res. 2004, 64, 423–428. 14729654 10.1158/0008-5472.can-03-2446

[38] Ström A; Hartman J; Foster JS; Kietz S; Wimalasena J; Gustafsson J-Å Estrogen receptor β inhibits 17β-estradiol-stimulated proliferation of the breast cancer cell line T47D. Proc. Natl. Acad. Sci. USA 2004, 101, 1566–1571. 14745018 10.1073/pnas.0308319100 PMC341775

[39] Lindberg K; Helguero LA; Omoto Y; Gustafsson J-Å; Haldosén L-A Estrogen receptor β represses Akt signaling in breast cancer cells via downregulation of HER2/HER3 and upregulation of PTEN: Implications for tamoxifen sensitivity. Breast Cancer Res. 2011, 13, R43. 21492444 10.1186/bcr2865 PMC3219206

[40] Cowley SM; Hoare S; Mosselman S; Parker MG Estrogen receptors α and β form heterodimers on DNA. J. Biol. Chem 1997, 272, 19858–19862. 9242648 10.1074/jbc.272.32.19858

[41] Pace P; Taylor J; Suntharalingam S; Coombes RC; Ali S Human estrogen receptor β binds DNA in a manner similar to and dimerizes with estrogen receptor α. J. Biol. Chem 1997, 272, 25832–25838. 9325313 10.1074/jbc.272.41.25832

[42] Pettersson K; Grandien K; Kuiper GG; Gustafsson J-A Mouse estrogen receptor β forms estrogen response element-binding heterodimers with estrogen receptor α. Mol. Endocrinol 1997, 11, 1486–1496. 9280064 10.1210/mend.11.10.9989

[43] Williams C; Edvardsson K; Lewandowski S; Ström A; Gustafsson J-Å A genome-wide study of the repressive effects of estrogen receptor beta on estrogen receptor alpha signaling in breast cancer cells. Oncogene 2008, 27, 1019–1032. 17700529 10.1038/sj.onc.1210712

[44] Grober O; Mutarelli M; Giurato G; Ravo M; Cicatiello L; De Filippo MR; Ferraro L; Nassa G; Papa MF; Paris O Global analysis of estrogen receptor beta binding to breast cancer cell genome reveals an extensive interplay with estrogen receptor alpha for target gene regulation. BMC Genom. 2011, 12, 36. 10.1186/1471-2164-12-36 PMC3025958 21235772

[45] Hartman J; Lindberg K; Morani A; Inzunza J; Strom A; Gustafsson J-A Estrogen receptor β inhibits angiogenesis and growth of T47D breast cancer xenografts. Cancer Res. 2006, 66, 11207–11213. 17145865 10.1158/0008-5472.CAN-06-0017

[46] Jonsson P; Katchy A; Williams C Support of a bi-faceted role of estrogen receptor beta (ERbeta) in ERalpha-positive breast cancer cells. Endocr. Relat. Cancer 2014, 21, 143–160. 24192230 10.1530/ERC-13-0444 PMC3946733

[47] Cheng J; Lee EJ; Madison LD; Lazennec G Expression of estrogen receptor β in prostate carcinoma cells inhibits invasion and proliferation and triggers apoptosis. FEBS Lett. 2004, 566, 169–172. 15147889 10.1016/j.febslet.2004.04.025

[48] Chaurasiya S; Wu W; Strom AM; Warner M; Gustafsson J-Å Estrogen receptor β regulates AKT activity through upregulation of INPP4B and inhibits migration of prostate cancer cell line PC-3. Proc. Natl. Acad. Sci. USA 2020, 117, 26347–26355. 33020300 10.1073/pnas.2007160117 PMC7584887

[49] Xiao L; Luo Y; Tai R; Zhang N Estrogen receptor β suppresses inflammation and the progression of prostate cancer. Mol. Med. Rep 2019, 19, 3555–3563. 30864712 10.3892/mmr.2019.10014 PMC6472045

[50] Mal R; Magner A; David J; Datta J; Vallabhaneni M; Kassem M; Manouchehri J; Willingham N; Stover D; Vandeusen J; Estrogen receptor beta (ERβ): A ligand activated tumor suppressor. Front. Oncol 2020, 10, 587386. 33194742 10.3389/fonc.2020.587386 PMC7645238

[51] Navaratnam S; Skliris G; Qing G; Banerji S; Badiani K; Tu D; Bradbury PA; Leighl NB; Shepherd FA; Nowatzki J; Differential role of estrogen receptor beta in early versus metastatic non-small cell lung cancer. Horm. Cancer 2012, 3, 93–100. 22302352 10.1007/s12672-012-0105-y PMC10358092

[52] Omoto Y; Kobayashi Y; Nishida K; Tsuchiya E; Eguchi H; Nakagawa K; Ishikawa Y; Yamori T; Iwase H; Fujii Y; Expression, function, and clinical implications of the estrogen receptor β in human lung cancers. Biochem. Biophys. Res. Commun 2001, 285, 340–347. 11444848 10.1006/bbrc.2001.5158

[53] Liu S; Hu C; Li M; An J; Zhou W; Guo J; Xiao Y Estrogen receptor beta promotes lung cancer invasion via increasing CXCR4 expression. Cell Death Dis. 2022, 13, 70. 35064116 10.1038/s41419-022-04514-4 PMC8782891

[54] Zhang G; Yanamala N; Lathrop KL; Zhang L; Klein-Seetharaman J; Srinivas H Ligand-independent antiapoptotic function of estrogen receptor-β in lung cancer cells. Mol. Endocrinol 2010, 24, 1737–1747. 20660297 10.1210/me.2010-0125 PMC2940472

[55] Mah V; Marquez D; Alavi M; Maresh EL; Zhang L; Yoon N; Horvath S; Bagryanova L; Fishbein MC; Chia D Expression levels of estrogen receptor beta in conjunction with aromatase predict survival in non-small cell lung cancer. Lung Cancer 2011, 74, 318–325. 21511357 10.1016/j.lungcan.2011.03.009 PMC3175023

[56] Hiramitsu S; Ishikawa T; Lee W-R; Khan T; Crumbley C; Khwaja N; Zamanian F; Asghari A; Sen M; Zhang Y Estrogen receptor beta-mediated modulation of lung cancer cell proliferation by 27-hydroxycholesterol. Front. Endocrinol 2018, 9, 470. 10.3389/fendo.2018.00470 PMC6116707 30190703

[57] Hershberger PA; Stabile LP; Kanterewicz B; Rothstein ME; Gubish CT; Land S; Shuai Y; Siegfried JM; Nichols M Estrogen receptor beta (ERβ) subtype-specific ligands increase transcription, p44/p42 mitogen activated protein kinase (MAPK) activation and growth in human non-small cell lung cancer cells. J. Steroid Biochem. Mol. Biol 2009, 116, 102–109. 19460433 10.1016/j.jsbmb.2009.05.004 PMC2722836

[58] Stabile LP; Dacic S; Land SR; Lenzner DE; Dhir R; Acquafondata M; Landreneau RJ; Grandis JR; Siegfried JM Combined analysis of estrogen receptor β−1 and progesterone receptor expression identifies lung cancer patients with poor outcome. Clin. Cancer Res 2011, 17, 154–164. 21062926 10.1158/1078-0432.CCR-10-0992 PMC3064257

[59] Yu W; Ding J; He M; Chen Y; Wang R; Han Z; Xing EZ; Zhang C; Yeh S Estrogen receptor β promotes the vasculogenic mimicry (VM) and cell invasion via altering the lncRNA-MALAT1/miR-145–5p/NEDD9 signals in lung cancer. Oncogene 2019, 38, 1225–1238. 30250297 10.1038/s41388-018-0463-1

[60] Song P; Li Y; Dong Y; Liang Y; Qu H; Qi D; Lu Y; Jin X; Guo Y; Jia Y; Estrogen receptor β inhibits breast cancer cells migration and invasion through CLDN6-mediated autophagy. J. Exp. Clin. Cancer Res 2019, 38, 354. 31412908 10.1186/s13046-019-1359-9 PMC6694553

[61] Thomas C; Rajapaksa G; Nikolos F; Hao R; Katchy A; McCollum CW; Bondesson M; Quinlan P; Thompson A; Krishnamurthy S; ERβ1 represses basal-like breast cancer epithelial to mesenchymal transition by destabilizing EGFR. Breast Cancer Res. 2012, 14, R148. 23158001 10.1186/bcr3358 PMC4053135

[62] Samanta S; Sharma VM; Khan A; Mercurio AM Regulation of IMP3 by EGFR signaling and repression by ERbeta: Implications for triple-negative breast cancer. Oncogene 2012, 31, 4689–4697. 22266872 10.1038/onc.2011.620 PMC3337950

[63] Lappano R; De Marco P; De Francesco EM; Chimento A; Pezzi V; Maggiolini M Cross-talk between GPER and growth factor signaling. J. Steroid Biochem. Mol. Biol 2013, 137, 50–56. 23542661 10.1016/j.jsbmb.2013.03.005

[64] Filardo EJ; Graeber CT; Quinn JA; Resnick MB; Giri D; DeLellis RA; Steinhoff MM; Sabo E Distribution of GPR30, a seven membrane–spanning estrogen receptor, in primary breast cancer and its association with clinicopathologic determinants of tumor progression. Clin. Cancer Res 2006, 12, 6359–6366. 17085646 10.1158/1078-0432.CCR-06-0860

[65] Thomas C; Karagounis IV; Srivastava RK; Vrettos N; Nikolos F; Francois N; Huang M; Gong S; Long Q; Kumar S Estrogen receptor β-mediated inhibition of actin-based cell migration suppresses metastasis of inflammatory breast cancer. Cancer Res. 2021, 81, 2399–2414. 33514514 10.1158/0008-5472.CAN-20-2743 PMC8570087

[66] Reese JM; Bruinsma ES; Nelson AW; Chernukhin I; Carroll JS; Li Y; Subramaniam M; Suman VJ; Negron V; Monroe DG ERβ-mediated induction of cystatins results in suppression of TGFβ signaling and inhibition of triple-negative breast cancer metastasis. Proc. Natl. Acad. Sci. USA 2018, 115, E9580–E9589. 30257941 10.1073/pnas.1807751115 PMC6187171

[67] Bouris P; Skandalis SS; Piperigkou Z; Afratis N; Karamanou K; Aletras AJ; Moustakas A; Theocharis AD; Karamanos NK Estrogen receptor alpha mediates epithelial to mesenchymal transition, expression of specific matrix effectors and functional properties of breast cancer cells. Matrix Biol. 2015, 43, 42–60. 25728938 10.1016/j.matbio.2015.02.008

[68] Sanchez AM; Flamini MI; Baldacci C; Goglia L; Genazzani AR; Simoncini T Estrogen receptor-α promotes breast cancer cell motility and invasion via focal adhesion kinase and N-WASP. Mol. Endocrinol 2010, 24, 2114–2125. 20880986 10.1210/me.2010-0252 PMC5417375

[69] Giretti MS; Fu X-D; De Rosa G; Sarotto I; Baldacci C; Garibaldi S; Mannella P; Biglia N; Sismondi P; Genazzani AR Extra-nuclear signalling of estrogen receptor to breast cancer cytoskeletal remodelling, migration and invasion. PLoS ONE 2008, 3, e2238. 18493596 10.1371/journal.pone.0002238 PMC2375059

[70] Dent R; Trudeau M; Pritchard KI; Hanna WM; Kahn HK; Sawka CA; Lickley LA; Rawlinson E; Sun P; Narod SA Triple-negative breast cancer: Clinical features and patterns of recurrence. Clin. Cancer Res 2007, 13, 4429–4434. 17671126 10.1158/1078-0432.CCR-06-3045

[71] Leung Y-K; Mak P; Hassan S; Ho S-M Estrogen receptor (ER)-β isoforms: A key to understanding ER-β signaling. Proc. Natl. Acad. Sci. USA 2006, 103, 13162–13167. 16938840 10.1073/pnas.0605676103 PMC1552044

[72] Wang J; Zhang C; Chen K; Tang H; Tang J; Song C; Xie X ERbeta1 inversely correlates with PTEN/PI3K/AKT pathway and predicts a favorable prognosis in triple-negative breast cancer. Breast Cancer Res. Treat 2015, 152, 255–269. 26070269 10.1007/s10549-015-3467-3

[73] Chantzi NI; Tiniakos DG; Palaiologou M; Goutas N; Filippidis T; Vassilaros SD; Dhimolea E; Mitsiou DJ; Alexis MN Estrogen receptor beta 2 is associated with poor prognosis in estrogen receptor alpha-negative breast carcinoma. J. Cancer Res. Clin. Oncol 2013, 139, 1489–1498. 23817696 10.1007/s00432-013-1467-4 PMC11824229

[74] Reese JM; Suman VJ; Subramaniam M; Wu X; Negron V; Gingery A; Pitel KS; Shah SS; Cunliffe HE; McCullough AE; ERbeta1: Characterization, prognosis, and evaluation of treatment strategies in ERalpha-positive and -negative breast cancer. BMC Cancer 2014, 14, 749. 25288324 10.1186/1471-2407-14-749 PMC4196114

[75] Aspros KG; Carter JM; Hoskin TL; Suman VJ; Subramaniam M; Emch MJ; Ye Z; Sun Z; Sinnwell JP; Thompson K Estrogen receptor beta repurposes EZH2 to suppress oncogenic NFκB/p65 signaling in triple negative breast cancer. NPJ Breast Cancer 2022, 8, 20. 35177654 10.1038/s41523-022-00387-0 PMC8854734

[76] Alexandrova E; Giurato G; Saggese P; Pecoraro G; Lamberti J; Ravo M; Rizzo F; Rocco D; Tarallo R; Nyman TA; Interaction proteomics identifies ERbeta association with chromatin repressive complexes to inhibit cholesterol biosynthesis and exert an oncosuppressive role in triple-negative breast cancer. Mol. Cell. Proteom 2020, 19, 245–260. 10.1074/mcp.RA119.001817 PMC7000115 31792072

[77] Yan S; Dey P; Ziegler Y; Jiao X; Kim SH; Katzenellenbogen JA; Katzenellenbogen BS Contrasting activities of estrogen receptor beta isoforms in triple negative breast cancer. Breast Cancer Res. Treat 2021, 185, 281–292. 33001337 10.1007/s10549-020-05948-0 PMC7867590

[78] Oturkar CC; Gandhi N; Rao P; Eng KH; Miller A; Singh PK; Zsiros E; Odunsi KO; Das GM Estrogen Receptor-Beta2 (ERbeta2)-Mutant p53-FOXM1 Axis: A Novel Driver of Proliferation, Chemoresistance, and Disease Progression in High Grade Serous Ovarian Cancer (HGSOC). Cancers 2022, 14, 1120. 35267428 10.3390/cancers14051120 PMC8909529

[79] Anestis A; Sarantis P; Theocharis S; Zoi I; Tryfonopoulos D; Korogiannos A; Koumarianou A; Xingi E; Thomaidou D; Kontos M; Estrogen receptor beta increases sensitivity to enzalutamide in androgen receptor-positive triple-negative breast cancer. J. Cancer Res. Clin. Oncol 2019, 145, 1221–1233. 30805773 10.1007/s00432-019-02872-9 PMC11810280

[80] Lei S; Fan P; Wang M; Zhang C; Jiang Y; Huang S; Fang M; He Z; Wu AJE; Medicine T Elevated estrogen receptor β expression in triple negative breast cancer cells is associated with sensitivity to doxorubicin by inhibiting the PI3K/AKT/mTOR signaling pathway. Exp. Ther. Med 2020, 20, 1630–1636. 32742395 10.3892/etm.2020.8809 PMC7388322

[81] Schüler-Toprak S; Häring J; Inwald EC; Moehle C; Ortmann O; Treeck O Agonists and knockdown of estrogen receptor β differentially affect invasion of triple-negative breast cancer cells in vitro. BMC Cancer 2016, 16, 951. 28003019 10.1186/s12885-016-2973-y PMC5178087

[82] Hinsche O; Girgert R; Emons G; Gründker C Estrogen receptor β selective agonists reduce invasiveness of triple-negative breast cancer cells. Int. J. Oncol 2015, 46, 878–884. 25420519 10.3892/ijo.2014.2778

[83] Andersson S; Sundberg M; Pristovsek N; Ibrahim A; Jonsson P; Katona B; Clausson C-M; Zieba A; Ramström M; Söderberg O Insufficient antibody validation challenges oestrogen receptor beta research. Nat. Commun 2017, 8, 15840. 28643774 10.1038/ncomms15840 PMC5501969

[84] Nelson AW; Groen AJ; Miller JL; Warren AY; Holmes KA; Tarulli GA; Tilley WD; Katzenellenbogen BS; Hawse JR; Gnanapragasam VJ; Comprehensive assessment of estrogen receptor beta antibodies in cancer cell line models and tissue reveals critical limitations in reagent specificity. Mol. Cell. Endocrinol 2017, 440, 138–150. 27889472 10.1016/j.mce.2016.11.016 PMC5228587

[85] Austin D; Hamilton N; Elshimali Y; Pietras R; Wu Y; Vadgama J Estrogen receptor-beta is a potential target for triple negative breast cancer treatment. Oncotarget 2018, 9, 33912. 30338035 10.18632/oncotarget.26089 PMC6188058

[86] Hamilton N; Márquez-Garbán D; Mah V; Fernando G; Elshimali Y; Garbán H; Elashoff D; Vadgama J; Goodglick L; Pietras R Biologic roles of estrogen receptor-β and insulin-like growth factor-2 in triple-negative breast cancer. BioMed Res. Int 2015, 2015, 925703. 25874233 10.1155/2015/925703 PMC4385615

[87] Paterni I; Granchi C; Katzenellenbogen JA; Minutolo F Estrogen receptors alpha (ERα) and beta (ERβ): Subtype-selective ligands and clinical potential. Steroids 2014, 90, 13–29. 24971815 10.1016/j.steroids.2014.06.012 PMC4192010

[88] Kumar R; Zakharov MN; Khan SH; Miki R; Jang H; Toraldo G; Singh R; Bhasin S; Jasuja R The dynamic structure of the estrogen receptor. J. Amino Acids 2011, 2011, 812540. 22312471 10.4061/2011/812540 PMC3268042

[89] Ruff M; Gangloff M; Marie Wurtz J; Moras D Estrogen receptor transcription and transactivation Structure-function relationship in DNA-and ligand-binding domains of estrogen receptors. Breast Cancer Res. 2000, 2, 353. 11250728 10.1186/bcr80 PMC138657

[90] Pike AC; Brzozowski AM; Hubbard RE; Bonn T; Thorsell A-G; Engström O; Ljunggren J; Gustafsson J-Å; Carlquist M Structure of the ligand-binding domain of oestrogen receptor beta in the presence of a partial agonist and a full antagonist. EMBO J. 1999, 18, 4608–4618. 10469641 10.1093/emboj/18.17.4608 PMC1171535

[91] Heldring N; Pike A; Andersson S; Matthews J; Cheng G; Hartman J; Tujague M; Strom A; Treuter E; Warner M Estrogen receptors: How do they signal and what are their targets. Physiol. Rev 2007, 87, 905–931. 17615392 10.1152/physrev.00026.2006

[92] Bryant HU; Glasebrook AL; Yang NN; Sato M An estrogen receptor basis for raloxifene action in bone. Proceedings of Xth International Congress on Hormonal Steroids, Quebec, Canada, 17–21 June 1998. J. Steroid Biochem. Mol. Biol 1999, 69, 37–44. 10418979 10.1016/s0960-0760(98)00147-2

[93] Hochner-Celnikier D Pharmacokinetics of raloxifene and its clinical application. Eur. J. Obstet. Gynecol. Reprod. Biol 1999, 85, 23–29. 10428318 10.1016/s0301-2115(98)00278-4

[94] Barrett-Connor E; Mosca L; Collins P; Geiger MJ; Grady D; Kornitzer M; McNabb MA; Wenger NK Effects of raloxifene on cardiovascular events and breast cancer in postmenopausal women. N. Engl. J. Med 2006, 355, 125–137. 16837676 10.1056/NEJMoa062462

[95] Grady D; Ettinger B; Moscarelli E; Plouffe L Jr.; Sarkar S; Ciaccia A; Cummings S; Multiple Outcomes of Raloxifene Evaluation Investigators. Safety and adverse effects associated with raloxifene: Multiple outcomes of raloxifene evaluation. Obstet. Gynecol 2004, 104, 837–844. 15458908 10.1097/01.AOG.0000137349.79204.b8

[96] Delmas PD; Bjarnason NH; Mitlak BH; Ravoux A-C; Shah AS; Huster WJ; Draper M; Christiansen C Effects of raloxifene on bone mineral density, serum cholesterol concentrations, and uterine endometrium in postmenopausal women. N. Engl. J. Med 1997, 337, 1641–1647. 9385122 10.1056/NEJM199712043372301

[97] Greish K; Nehoff H; Bahman F; Pritchard T; Taurin S Raloxifene nano-micelles effect on triple-negative breast cancer is mediated through estrogen receptor-β and epidermal growth factor receptor. J. Drug Target 2019, 27, 903–916. 30615483 10.1080/1061186X.2019.1566341

[98] Matsushima-Nishiwaki R; Yamada N; Hattori Y; Hosokawa Y; Tachi J; Hori T; Kozawa O SERMs (selective estrogen receptor modulator), acting as estrogen receptor β agonists in hepatocellular carcinoma cells, inhibit the transforming growth factor-α-induced migration via specific inhibition of AKT signaling pathway. PLoS ONE 2022, 17, e0262485. 35007301 10.1371/journal.pone.0262485 PMC8746762

[99] Pozios I; Seel NN; Hering NA; Hartmann L; Liu V; Camaj P; Müller MH; Lee LD; Bruns CJ; Kreis ME Raloxifene inhibits pancreatic adenocarcinoma growth by interfering with ERβ and IL-6/gp130/STAT3 signaling. Cell. Oncol 2021, 44, 167–177. 10.1007/s13402-020-00559-9 PMC7906944 32940862

[100] Palmer H; Nimick M; Mazumder A; Taurin S; Rana Z; Rosengren R Raloxifene Suppresses Tumor Growth and Metastasis in an Orthotopic Model of Castration-Resistant Prostate Cancer. Biomedicines 2022, 10, 853. 35453603 10.3390/biomedicines10040853 PMC9033055

[101] Love RR; Mazess RB; Barden HS; Epstein S; Newcomb PA; Jordan VC; Carbone PP; DeMets DL Effects of tamoxifen on bone mineral density in postmenopausal women with breast cancer. N. Engl. J. Med 1992, 326, 852–856. 1542321 10.1056/NEJM199203263261302

[102] Kedar R; Bourne TH; Collins W; Campbell S; Powles T; Ashley S; Cosgrove D Effects of tamoxifen on uterus and ovaries of postmenopausal women in a randomised breast cancer prevention trial. Lancet 1994, 343, 1318–1321. 7910323 10.1016/s0140-6736(94)92466-x

[103] Osborne CK Tamoxifen in the treatment of breast cancer. N. Engl. J. Med 1998, 339, 1609–1618. 9828250 10.1056/NEJM199811263392207

[104] Early Breast Cancer Trialists’ Collaborative Group. Effects of chemotherapy and hormonal therapy for early breast cancer on recurrence and 15-year survival: An overview of the randomised trials. Lancet 2005, 365, 1687–1717. 15894097 10.1016/S0140-6736(05)66544-0

[105] Esslimani-Sahla M; Simony-Lafontaine J; Kramar A; Lavaill R; Mollevi C; Warner M; Gustafsson J.-A.k.; Rochefort H Estrogen receptor β (ERβ) level but not its ERβcx variant helps to predict tamoxifen resistance in breast cancer. Clin. Cancer Res 2004, 10, 5769–5776. 15355905 10.1158/1078-0432.CCR-04-0389

[106] Hopp TA; Weiss HL; Parra IS; Cui Y; Osborne CK; Fuqua SA Low levels of estrogen receptor β protein predict resistance to tamoxifen therapy in breast cancer. Clin. Cancer Res 2004, 10, 7490–7499. 15569979 10.1158/1078-0432.CCR-04-1114

[107] Lattrich C; Schüler S; Häring J; Skrzypczak M; Ortmann O; Treeck O Effects of a combined treatment with tamoxifen and estrogen receptor β agonists on human breast cancer cell lines. Arch. Gynecol. Obstet 2014, 289, 163–171. 23907354 10.1007/s00404-013-2977-7

[108] Hodges-Gallagher L; Valentine CD; Bader SE; Kushner P Estrogen receptor beta increases the efficacy of antiestrogens by effects on apoptosis and cell cycling in breast cancer cells. Breast Cancer Res. 2008, 109, 241–250. 10.1007/s10549-007-9640-6 17638070

[109] Rajapaksa G; Nikolos F; Bado I; Clarke R; Gustafsson J-Å; Thomas C ERβ decreases breast cancer cell survival by regulating the IRE1/XBP-1 pathway. Oncogene 2015, 34, 4130–4141. 25347741 10.1038/onc.2014.343

[110] Razandi M; Pedram A; Jordan VC; Fuqua S; Levin ER Tamoxifen regulates cell fate through mitochondrial estrogen receptor beta in breast cancer. Oncogene 2013, 32, 3274–3285. 22907432 10.1038/onc.2012.335 PMC3505272

[111] Langendonk M; de Jong MR; Smit N; Seiler J; Reitsma B; Ammatuna E; Glaudemans AW; van den Berg A; Huls GA; Visser L Identification of the estrogen receptor beta as a possible new tamoxifen-sensitive target in diffuse large B-cell lymphoma. Blood Cancer J. 2022, 12, 36. 35256592 10.1038/s41408-022-00631-7 PMC8901714

[112] Zhao L; Huang S; Mei S; Yang Z; Xu L; Zhou N; Yang Q; Shen Q; Wang W; Le X; Pharmacological activation of estrogen receptor beta augments innate immunity to suppress cancer metastasis. Proc. Natl. Acad. Sci. USA 2018, 115, E3673–E3681. 29592953 10.1073/pnas.1803291115 PMC5910874

[113] Huang S; Zhou N; Zhao L; Gimple RC; Ahn YH; Zhang P; Wang W; Shao B; Yang J; Zhang Q Pharmacological activation of estrogen receptor beta overcomes tumor resistance to immune checkpoint blockade therapy. IScience 2020, 23, 101458. 32861994 10.1016/j.isci.2020.101458 PMC7476860

[114] He Y; Alejo S; Venkata PP; Johnson JD; Loeffel I; Pratap UP; Zou Y; Lai Z; Tekmal RR; Kost ER Therapeutic targeting of ovarian Cancer stem cells using estrogen receptor Beta agonist. Int. J. Mol. Sci 2022, 23, 7159. 35806169 10.3390/ijms23137159 PMC9266546

[115] Sareddy GR; Li X; Liu J; Viswanadhapalli S; Garcia L; Gruslova A; Cavazos D; Garcia M; Strom AM; Gustafsson J-A Selective estrogen receptor β agonist LY500307 as a novel therapeutic agent for glioblastoma. Sci. Rep 2016, 6, 24185. 27126081 10.1038/srep24185 PMC4850367

[116] Pratap UP; Sareddy GR; Liu Z; Venkata PP; Liu J; Tang W; Altwegg KA; Ebrahimi B; Li X; Tekmal RR Histone deacetylase inhibitors enhance estrogen receptor beta expression and augment agonist-mediated tumor suppression in glioblastoma. Neuro-Oncol. Adv 2021, 3, vdab099. 10.1093/noajnl/vdab099 PMC8412056 34485908

[117] Pontecorvi G; Bellenghi M; Tait S; Tirelli V; Matarrese P; Mattia G; Carè A; Puglisi R Different Susceptibilities of Human Melanoma Cell Lines to G2/M Blockage and Cell Death Activation in Response to the Estrogen Receptor β agonist LY500307. J. Cancer 2022, 13, 1573. 35371312 10.7150/jca.65425 PMC8965114

[118] Roehrborn C; Spann M; Myers S; Serviss C; Hu L; Jin Y Estrogen receptor beta agonist LY500307 fails to improve symptoms in men with enlarged prostate secondary to benign prostatic hypertrophy. Prostate Cancer Prostatic Dis. 2015, 18, 43–48. 25348255 10.1038/pcan.2014.43

[119] Breier A The Efficacy and Safety of a Selective Estrogen Receptor Beta Agonist (LY500307) for Negative Symptoms and Cognitive Impairment Associated With Schizophrenia—Full Text View—ClinicalTrials. gov. 2019.

[120] Shen SS; Smith CL; Hsieh JT; Yu J; Kim IY; Jian W; Sonpavde G; Ayala GE; Younes M; Lerner SP Expression of estrogen receptors-α and-β in bladder cancer cell lines and human bladder tumor tissue. Interdiscip. Int. J. Am. Cancer Soc 2006, 106, 2610–2616. 10.1002/cncr.21945 16700038

[121] Kauffman EC; Robinson BD; Downes M; Marcinkiewicz K; Vourganti S; Scherr DS; Gudas LJ; Mongan NP Estrogen receptor-β expression and pharmacological targeting in bladder cancer. Oncol. Rep 2013, 30, 131–138. 23612777 10.3892/or.2013.2416 PMC3729232

[122] Rao Q; Chen Y; Yeh C-R; Ding J; Li L; Chang C; Yeh S Recruited mast cells in the tumor microenvironment enhance bladder cancer metastasis via modulation of ERβ/CCL2/CCR2 EMT/MMP9 signals. Oncotarget 2016, 7, 7842. 26556868 10.18632/oncotarget.5467 PMC4884958

[123] Tao L; Qiu J; Slavin S; Ou Z; Liu Z; Ge J; Zuo L; Guancial EA; Messing EM; Chang C Recruited T cells promote the bladder cancer metastasis via up-regulation of the estrogen receptor β/IL-1/c-MET signals. Cancer Lett. 2018, 430, 215–223. 29684419 10.1016/j.canlet.2018.03.045

[124] Ali HR; Chlon L; Pharoah PD; Markowetz F; Caldas C Patterns of immune infiltration in breast cancer and their clinical implications: A gene-expression-based retrospective study. PLoS Med. 2016, 13, e1002194. 27959923 10.1371/journal.pmed.1002194 PMC5154505

[125] Onesti CE; Josse C; Poncin A; Frères P; Poulet C; Bours V; Jerusalem G Predictive and prognostic role of peripheral blood eosinophil count in triple-negative and hormone receptor-negative/HER2-positive breast cancer patients undergoing neoadjuvant treatment. Oncotarget 2018, 9, 33719. 30263098 10.18632/oncotarget.26120 PMC6154746

[126] Ghebeh H; Elshenawy MA; AlSayed AD; Al-Tweigeri T Peripheral blood eosinophil count is associated with response to chemoimmunotherapy in metastatic triple-negative breast cancer. Immunotherapy 2022, 14, 189–199. 34984928 10.2217/imt-2021-0149

[127] Artham S; Chang C-Y; McDonnell DP Eosinophilia in cancer and its regulation by sex hormones. Trends Endocrinol. Metab 2023, 34, 5–20. 36443206 10.1016/j.tem.2022.11.002 PMC10122120

[128] Polanczyk MJ; Carson BD; Subramanian S; Afentoulis M; Vandenbark AA; Ziegler SF; Offner H Cutting edge: Estrogen drives expansion of the CD4+ CD25+ regulatory T cell compartment. J. Immunol 2004, 173, 2227–2230. 15294932 10.4049/jimmunol.173.4.2227

[129] Polanczyk MJ; Hopke C; Vandenbark AA; Offner H Treg suppressive activity involves estrogen-dependent expression of programmed death-1 (PD-1). Int. Immunol 2007, 19, 337–343. 17267414 10.1093/intimm/dxl151

[130] Edvardsson K; Ström A; Jonsson P; Gustafsson J-Å; Williams C Estrogen receptor β induces antiinflammatory and antitumorigenic networks in colon cancer cells. Mol. Endocrinol 2011, 25, 969–979. 21493669 10.1210/me.2010-0452 PMC5417254

[131] Campbell L; Emmerson E; Davies F; Gilliver SC; Krust A; Chambon P; Ashcroft GS; Hardman M Estrogen promotes cutaneous wound healing via estrogen receptor β independent of its antiinflammatory activities. J. Exp. Med 2010, 207, 1825–1833. 20733032 10.1084/jem.20100500 PMC2931162

[132] Sun J; Ma X; Chen Y-X; Rayner K; Hibbert B; McNulty M; Dhaliwal B; Simard T; Ramirez D; O’Brien E Attenuation of atherogenesis via the anti-inflammatory effects of the selective estrogen receptor beta modulator 8β-VE2. J. Cardiovasc. Pharmacol 2011, 58, 399–405. 21697723 10.1097/FJC.0b013e318226bd16

[133] Xing D; Feng W; Miller AP; Weathington NM; Chen Y-F; Novak L; Blalock JE; Oparil S; Physiology C Estrogen modulates TNF-α-induced inflammatory responses in rat aortic smooth muscle cells through estrogen receptor-β activation. Am. J. Physiol.-Heart Circ. Physiol 2007, 292, H2607–H2612. 17237237 10.1152/ajpheart.01107.2006

[134] Wang L; Qiu X; Hao Q; Li D Anti-inflammatory effects of a Chinese herbal medicine in atherosclerosis via estrogen receptor β mediating nitric oxide production and NF-κB suppression in endothelial cells. Cell Death Dis. 2013, 4, e551. 23519120 10.1038/cddis.2013.66 PMC3615733

[135] Guo D; Liu X; Zeng C; Cheng L; Song G; Hou X; Zhu L; Zou K Estrogen receptor β activation ameliorates DSS-induced chronic colitis by inhibiting inflammation and promoting Treg differentiation. Int. Immunopharmacol 2019, 77, 105971. 31678865 10.1016/j.intimp.2019.105971

[136] Pierdominici M; Maselli A; Varano B; Barbati C; Cesaro P; Spada C; Zullo A; Lorenzetti R; Rosati M; Rainaldi G Linking estrogen receptor β expression with inflammatory bowel disease activity. Oncotarget 2015, 6, 40443. 26497217 10.18632/oncotarget.6217 PMC4747344

[137] Yuan B; Clark CA; Wu B; Yang J; Drerup JM; Li T; Jin VX; Hu Y; Curiel TJ; Li R Estrogen receptor beta signaling in CD8(+) T cells boosts T cell receptor activation and antitumor immunity through a phosphotyrosine switch. J. Immunother. Cancer 2021, 9, e001932. 33462142 10.1136/jitc-2020-001932 PMC7816924

[138] Sapino A; Bosco M; Cassoni P; Castellano I; Arisio R; Cserni G; Dei Tos AP; Fortunati N; Catalano MG; Bussolati G Estrogen receptor-beta is expressed in stromal cells of fibroadenoma and phyllodes tumors of the breast. Mod. Pathol 2006, 19, 599–606. 16554735 10.1038/modpathol.3800574

[139] Shim GJ; Gherman D; Kim HJ; Omoto Y; Iwase H; Bouton D; Kis LL; Andersson CT; Warner M; Gustafsson JA Differential expression of oestrogen receptors in human secondary lymphoid tissues. J. Pathol 2006, 208, 408–414. 16294372 10.1002/path.1883

[140] Pierdominici M; Maselli A; Colasanti T; Giammarioli AM; Delunardo F; Vacirca D; Sanchez M; Giovannetti A; Malorni W; Ortona E Estrogen receptor profiles in human peripheral blood lymphocytes. Immunol. Lett 2010, 132, 79–85. 20542061 10.1016/j.imlet.2010.06.003

[141] Bukovsky A; Caudle MR; Cekanova M; Fernando RI; Wimalasena J; Foster JS; Henley DC; Elder RF Placental expression of estrogen receptor beta and its hormone binding variant--comparison with estrogen receptor alpha and a role for estrogen receptors in asymmetric division and differentiation of estrogen-dependent cells. Reprod. Biol. Endocrinol 2003, 1, 36. 12740031 10.1186/1477-7827-1-36 PMC155643

